# Translational insights into manufacturability and stability of broadly neutralizing antibodies for pediatric HIV prevention: lessons from plant-produced CAP256-VRC26.25

**DOI:** 10.3389/fped.2026.1837801

**Published:** 2026-08-10

**Authors:** Tsepo L. Tsekoa, Lusisizwe Kwezi, Priyen Pillay, Sibongile Mtimka, Maabo Moralo, Kabamba Alexandre, Joseph Nkolola, Dan H. Barouch, Rachel Chikwamba

**Affiliations:** 1 Future Production: Chemicals Cluster, Council for Scientific and Industrial Research, Pretoria, South Africa; 2 Center for Virology and Vaccine Research, Beth Israel Deaconess Medical Center, Boston, MA, United States; 3 Harvard Medical School, Boston, MA, United States

**Keywords:** antibody manufacturability, broadly neutralizing antibodies, hiv prevention, pharmacokinetics, plant molecular farming

## Abstract

Monoclonal antibodies hold significant promise for preventing HIV infection in infants and children. However, global access remains constrained by the high production costs and infrastructure requirements associated with complex mammalian cell manufacturing platforms. Alternative expression systems, including plant-based production, have been proposed as scalable and potentially lower-cost approaches for antibody manufacturing. Here, we evaluated the *in vivo* performance of plant-produced CAP256-VRC26.25, a potent V2-apex HIV-1 broadly neutralizing antibody originally isolated from an HIV-infected individual in South Africa. Purified, endotoxin-free plant-produced CAP256-VRC26.25 was administered to cynomolgus macaques alongside a mammalian cell-produced CAP256-VRC26.25 control antibody prior to mucosal SHIV challenge. While the mammalian-derived antibody conferred protection, the plant-produced antibody did not. Pharmacokinetic analysis revealed approximately two orders of magnitude lower circulating antibody levels and rapid clearance of the plant-produced antibody, despite preserved *in vitro* neutralization potency. Electrophoretic analysis indicated evidence of partial proteolytic nicking of the plant-produced antibody, suggesting that structural instability may have contributed to reduced *in vivo* durability. Previous studies have shown that manufacturability liabilities within the CAP256-VRC26 lineage can occur across multiple expression platforms, highlighting the importance of integrating antibody engineering with manufacturing platform development. These findings have informed ongoing work combining host genome engineering to reduce endogenous plant protease activity with targeted modification of predicted protease-sensitive sites within CAP256-VRC26.25. Overall, this study provides translational insights into the engineering challenges associated with scalable production of broadly neutralizing antibodies and highlights key considerations for developing accessible antibody-based interventions for pediatric HIV prevention.

## 1 Introduction

Despite substantial progress in the prevention and treatment of HIV infection, children remain disproportionately affected in many parts of the world. In 2023, an estimated 1.4 million children were living with HIV globally, with the majority residing in sub-Saharan Africa (1). While the scale-up of antiretroviral therapy and programs for the prevention of mother-to-child transmission have significantly reduced pediatric infections (2, 3), gaps in maternal diagnosis, treatment adherence, and health system coverage continue to result in new infections among infants and young children (4). Additional preventive strategies that complement antiretroviral approaches are therefore needed, particularly in settings where access to consistent care remains challenging (5–7).

Broadly neutralizing antibodies (bnAbs) directed against the HIV envelope glycoprotein have emerged as promising candidates for both prevention and treatment (8, 9). These antibodies recognize conserved epitopes across diverse viral strains and have demonstrated potent antiviral activity in preclinical models and early clinical studies (8). Proof-of-concept for antibody-mediated HIV prevention was established in the Antibody Mediated Prevention (AMP) trials, which demonstrated that the CD4-binding site bnAb VRC01 could prevent acquisition of HIV strains sensitive to the antibody (10, 11). Long-acting variants engineered with Fc modifications have further extended serum half-life, supporting the possibility of infrequent dosing for prophylactic use (12, 13). In pediatric contexts, bnAbs could complement existing prevention strategies by providing passive immunity to infants during periods of highest transmission risk, including the perinatal and breastfeeding periods (14). Such approaches may offer additional protection in situations where maternal antiretroviral therapy is delayed, interrupted, or insufficient to fully prevent transmission (15, 16).

More recently, clinical studies have begun to evaluate bnAbs in pediatric contexts. Early trials have shown that bnAbs such as VRC01 can be safely administered to HIV-exposed newborn infants, supporting their potential use for prevention of vertical transmission (17). In parallel, long-acting bnAb variants including VRC07-523LS are being investigated for infant prophylaxis, and combination bnAb regimens are being evaluated in children living with HIV as part of remission or cure strategies (18). These developments highlight the growing clinical interest in antibody-based interventions for pediatric HIV prevention and treatment.

However, the translation of monoclonal antibody interventions to pediatric populations faces significant challenges. Chief among these are the high costs and complex infrastructure associated with conventional mammalian-cell manufacturing platforms, which remain the dominant method for producing therapeutic antibodies (19, 20). These systems rely on large-scale bioreactor facilities, extensive purification processes, and highly specialized supply chains, all of which contribute to the high cost of antibody medicines (19). As a result, most monoclonal antibodies remain financially inaccessible for large-scale use in pediatric populations in low- and middle-income countries where the burden of HIV and other infectious diseases is greatest (21, 22). Developing innovative scalable manufacturing approaches that can reduce costs while maintaining product quality is therefore particularly important for expanding global access to antibody-based prevention strategies (23, 24).

Plant-based expression systems have been explored as an alternative platform to produce recombinant proteins and monoclonal antibodies (25–28). Transient expression in *Nicotiana benthamiana* has emerged as a flexible and scalable approach capable of rapidly generating complex biologics without the need for stable cell line development (29, 30). Plant molecular farming has been used successfully to produce vaccines, enzymes, and antibodies (27, 31), and offers potential advantages in terms of manufacturing speed, infrastructure requirements, and decentralized production (31, 32). Nevertheless, plant-derived antibodies can face specific technical challenges, including differences in glycosylation patterns and susceptibility to proteolytic degradation during expression, which may influence their stability and pharmacokinetic properties (33, 34).

CAP256-VRC26.25 is a potent HIV-1 broadly neutralizing antibody targeting the V2 apex of the envelope glycoprotein (35, 36). The CAP256-VRC26 antibody lineage was originally isolated from a South African individual who developed exceptional neutralizing breadth during natural infection (35). Because of its potency and breadth, CAP256-VRC26.25 has been considered a promising candidate for preventive applications, including maternal-infant HIV transmission settings (36). V2-apex bnAbs such as CAP256-VRC26.25 exhibit remarkable neutralization breadth and potency against diverse HIV-1 strains (36). Developing scalable manufacturing approaches for such antibodies is therefore particularly important for expanding access to bnAb-based prevention strategies in regions most affected by pediatric HIV.

In this study, we evaluated the prophylactic efficacy and pharmacokinetic performance of plant-produced CAP256-VRC26.25 in a cynomolgus macaque SHIV challenge model, comparing it directly with mammalian-derived antibody (36, 37). Purified plant-derived CAP256-VRC26.25 was assessed for its ability to confer protection against viral challenge and for its *in vivo* stability following administration. Although the plant-produced antibody retained *in vitro* neutralization potency, *in vivo* evaluation revealed markedly reduced serum exposure and rapid clearance. Electrophoretic analysis suggested that partial proteolytic nicking during plant expression may have contributed to these effects. By analyzing the translational performance of this plant-derived bnAb, we identify key manufacturability and stability constraints that have informed subsequent host- and antibody-engineering strategies for developing scalable and affordable antibody-based prevention approaches for children.

## 2 Manufacturing platforms for monoclonal antibodies and the value proposition of plant-based systems

Monoclonal antibodies are predominantly manufactured using mammalian cell culture systems, most commonly Chinese hamster ovary (CHO) cells, which have become the industry standard for therapeutic antibody production. These platforms are capable of producing large quantities of antibodies with appropriate post-translational modifications and well-established regulatory frameworks (38). Over the past three decades, advances in cell line engineering, media optimization, and downstream purification have significantly improved antibody production yields and process robustness (39, 40). Modern fed-batch and continuous CHO manufacturing processes achieve major gains in productivity and manufacturing efficiency (23, 41).

At the same time, mammalian cell manufacturing remains capital intensive and technologically complex, requiring large-scale bioreactors, specialized infrastructure, and highly controlled production environments. These requirements contribute to the high cost of goods associated with many antibody therapeutics and can constrain global manufacturing capacity (42). The growing interest in monoclonal antibodies for infectious disease prevention, including applications in pediatric populations, has therefore stimulated exploration of alternative production platforms that may offer complementary advantages in scalability, cost, or deployment flexibility. Manufacturing innovation has increasingly been recognized as a critical determinant of the global accessibility of antibody-based interventions, particularly in low- and middle-income countries where infrastructure and supply constraints can limit availability (23).

In response to these challenges, a range of alternative expression systems has been investigated, including microbial hosts, transgenic animals, cell-free expression systems, and plant-based molecular farming. Each platform presents distinct advantages and limitations in terms of productivity, product quality, scalability, and regulatory maturity. Plant-based expression systems have attracted particular attention as flexible platforms for recombinant protein production. Transient expression in *Nicotiana benthamiana* enables rapid production of recombinant proteins without the need to generate stable cell lines, allowing candidate molecules to be produced within weeks following sequence design. Plant systems can also be scaled through relatively facile cultivation rather than large bioreactors, potentially reducing upstream production infrastructure requirements and enabling more geographically distributed production models.

The potential economic advantages of plant-based antibody production have been explored through technoeconomic modelling studies. Nandi et al. demonstrated that plant molecular farming could offer competitive production costs for monoclonal antibodies under certain production scenarios, particularly at moderate scales and in contexts where rapid or flexible manufacturing capacity is desirable (43). However, the economic landscape of antibody manufacturing continues to evolve rapidly. Advances in mammalian cell culture, including the development of high-producing CHO cell lines and the increasing adoption of continuous and intensified bioprocessing strategies, have substantially improved productivity and reduced cost of goods for conventional antibody production. Emerging continuous manufacturing approaches being developed by groups such as SevareGMP and industrial manufacturers including Enzene Biosciences illustrate how process intensification and modular facility designs may enable more efficient and geographically distributed antibody manufacturing using mammalian systems. In this context, plant molecular farming should not necessarily be viewed as a direct replacement for established and innovative mammalian production platforms. Rather, plant-based systems may offer complementary advantages in specific scenarios, including rapid response manufacturing, decentralized production models, or the production of complex antibody formats that are difficult to assemble efficiently in conventional expression hosts.

Beyond potential economic benefits, plant systems may therefore be well suited to the production of complex antibody architectures that require orchestrated assembly of multiple polypeptide components. Multimeric antibodies such as secretory immunoglobulin A (sIgA), for example, require the coordinated expression of heavy chains, light chains, joining chains, and secretory components. The structural complexity of sIgA has made recombinant production challenging across expression platforms, with correct multichain assembly, purification, yield, and stability representing important barriers to scalable therapeutic development (44). Studies led by Julian Ma and colleagues have demonstrated that plant expression systems can support the efficient assembly and production of functional sIgA molecules with appropriate structural properties (45, 46). Enhancements in the quality and yield of recombinant sIgA antibodies have been achieved in plants through endoplasmic reticulum engineering (47). These antibody formats are of particular interest for pediatric applications because secretory antibodies play a central role in mucosal immunity at gastrointestinal, respiratory, and reproductive surfaces, which represent important sites of pathogen exposure in infants and young children, including breastfeeding-associated HIV exposure. In this context, mucosal antibody formats represent a broader translational pediatric opportunity for plant-based production platforms, alongside the systemic IgG approach evaluated here.

Despite these advantages, plant-based antibody production also presents technical challenges that must be addressed to achieve robust and scalable manufacturing. Host-specific factors, including endogenous proteases, differences in post-translational processing, and potential impacts on protein stability, can influence the quality and performance of recombinant proteins produced in plant systems. Addressing these issues requires careful optimization of both the host expression system and the antibody molecule itself.

Within this broader manufacturing landscape, broadly neutralizing antibodies directed against HIV provide a useful case study for examining how production platform characteristics can influence antibody stability, manufacturability, and translational potential. CAP256-VRC26.25 is a potent V2-apex broadly neutralizing antibody originally isolated from an HIV-infected donor in the CAPRISA cohort and subsequently characterized as part of the CAP256-VRC26 antibody lineage (48, 49). As interest grows in deploying bnAbs for prevention of HIV infection, including in infants and other vulnerable populations, understanding how different manufacturing platforms affect antibody quality and *in vivo* performance becomes increasingly important. Evaluating the production and behavior of CAP256-VRC26.25 in alternative expression systems therefore provides an opportunity to explore both the potential and the engineering challenges associated with developing scalable manufacturing strategies for bnAb-based interventions.

## 3 Results

### 3.1 Production and characterization of plant-derived CAP256-VRC26.25

The HIV-1 broadly neutralizing antibody CAP256-VRC26.25 used in this study was produced using a transient expression system in *Nicotiana benthamiana*. The plant expression platform and associated host engineering strategies have been described previously (26, 50). Fully assembled CAP256-VRC26.25 IgG was successfully produced and purified from infiltrated plant tissue to generate endotoxin-free antibody suitable for *in vivo* studies. Initial analytical characterization by non-reducing SDS-PAGE and SEC-HPLC (Supplementary Figure S1A,B) supported the presence of a major intact antibody species with the expected heavy- and light-chain composition. *In vitro* neutralization assays further confirmed that the plant-produced antibody retained the expected neutralizing activity prior to evaluation in the non-human primate challenge model. Purified CAP256-VRC26.25 mAbs samples contained 0.12 EU/mL of endotoxins. The same purified antibody batch was consistently used for the non-human primate study and analytical investigations.

### 3.2 *In vivo* evaluation and pharmacokinetic behavior of CAP256-VRC26.25

The prophylactic efficacy of plant-produced CAP256-VRC26.25 was evaluated in a small exploratory non-human primate study using a mucosal SHIV challenge system that has been widely used to assess the protective activity of HIV broadly neutralizing antibodies (36, 37, 51). Cynomolgus macaques (*Macaca fascicularis*) were used to evaluate the ability of CAP256-VRC26.25 to protect against mucosal viral exposure using the SHIV-325CH strain.

**Figure 1:**
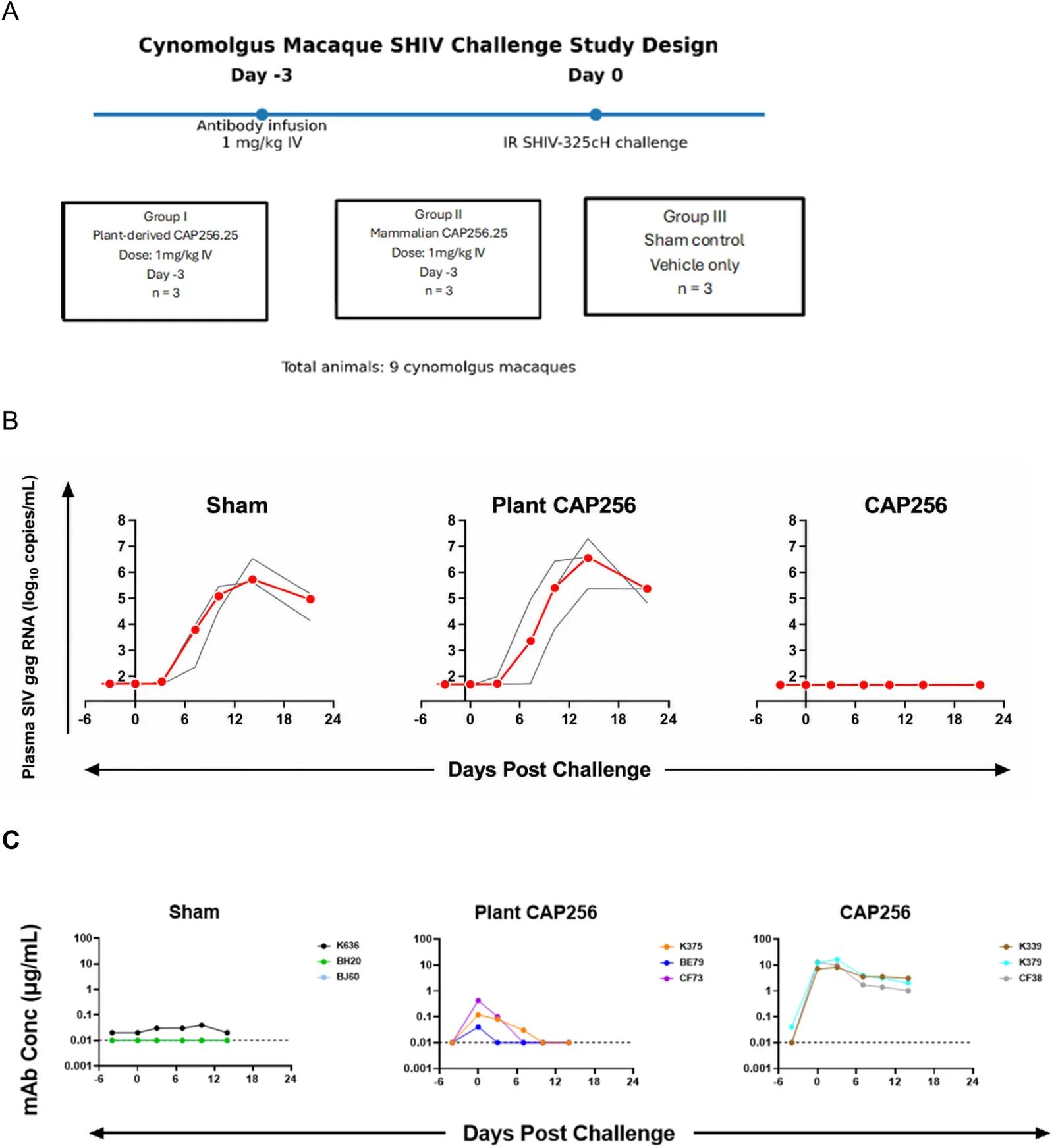
Non-human primate study design, infection outcomes, and pharmacokinetics. **(A)** Study design showing antibody administration and SHIV-325CH challenge. **(B)** Plasma SIV gag RNA levels over time following challenge, shown as log10 copies/mL. **(C)** Pharmacokinetic comparison of plant-produced and mammalian-derived CAP256-VRC26.25.

Animals were assigned to three experimental groups (*n* = 3 per group). One group received plant-derived CAP256-VRC26.25, a second group received CAP256-VRC26.25 produced using a mammalian expression system as a positive control, and a third group received sham treatment (Figure 1A). Antibodies were administered intravenously at a concentration of 1 mg/kg. Three days following antibody infusion, all animals were challenged intrarectally with SHIV-325CH. Consistent with previous studies demonstrating the protective activity of broadly neutralizing antibodies in this model (36), animals receiving mammalian-derived CAP256-VRC26.25 were protected against viral challenge under the conditions tested. In contrast, animals receiving plant-derived CAP256-VRC26.25 did not exhibit comparable protection following SHIV challenge, and infection outcomes in this group were similar to those observed in sham-treated animals (Figure 1B).

To further investigate the basis for the lack of protection observed with the plant-derived antibody, serum antibody concentrations were measured following administration. Animals that received mammalian-derived CAP256-VRC26.25 exhibited circulating antibody levels consistent with previously reported pharmacokinetic profiles of broadly neutralizing antibodies in non-human primates (36, 37, 51). In contrast, animals receiving plant-derived CAP256-VRC26.25 displayed markedly reduced circulating antibody concentrations. Peak serum levels of the plant-produced antibody were approximately two orders of magnitude lower than those observed with the mammalian-derived antibody and declined rapidly following administration, indicating substantially faster clearance kinetics (Figure 1C).

The observed differences between the plant-produced and mammalian-derived CAP256-VRC26.25 preparations were found to be consistent and pronounced across multiple readouts, including serum antibody levels, viral outcomes, and protection following SHIV challenge. These findings suggested that the plant-derived CAP256-VRC26.25 antibody experienced reduced *in vivo* stability compared with the mammalian-produced antibody. Because *in vitro* neutralization assays performed prior to the animal study confirmed that the plant-derived antibody retained the expected neutralizing activity, the reduced protective efficacy observed *in vivo* was unlikely to reflect loss of antigen-binding function. Instead, the markedly reduced systemic exposure of the plant-derived antibody suggested that structural or biochemical differences affecting antibody stability may have influenced its pharmacokinetic behavior. This observation prompted further analytical investigation of the structural integrity of the plant-produced CAP256-VRC26.25 preparation.

### 3.3 Analytical investigation of reduced *in vivo* stability

To further investigate the basis for the rapid clearance observed for plant-derived CAP256-VRC26.25 *in vivo*, additional analytical characterization of the antibody preparation was performed. Initial quality control analyses conducted prior to the non-human primate study indicated that the purified antibody retained the expected quaternary structure of an assembled IgG molecule, as confirmed by electrophoretic and chromatographic analyses of the same batch under non-reducing conditions (Supplementary Figure S1A,B). These results suggested that the antibody preparation contained predominantly intact heavy and light chain assemblies and did not exhibit substantial aggregation or fragmentation at the level detectable by these analytical methods.

**Figure 2:**
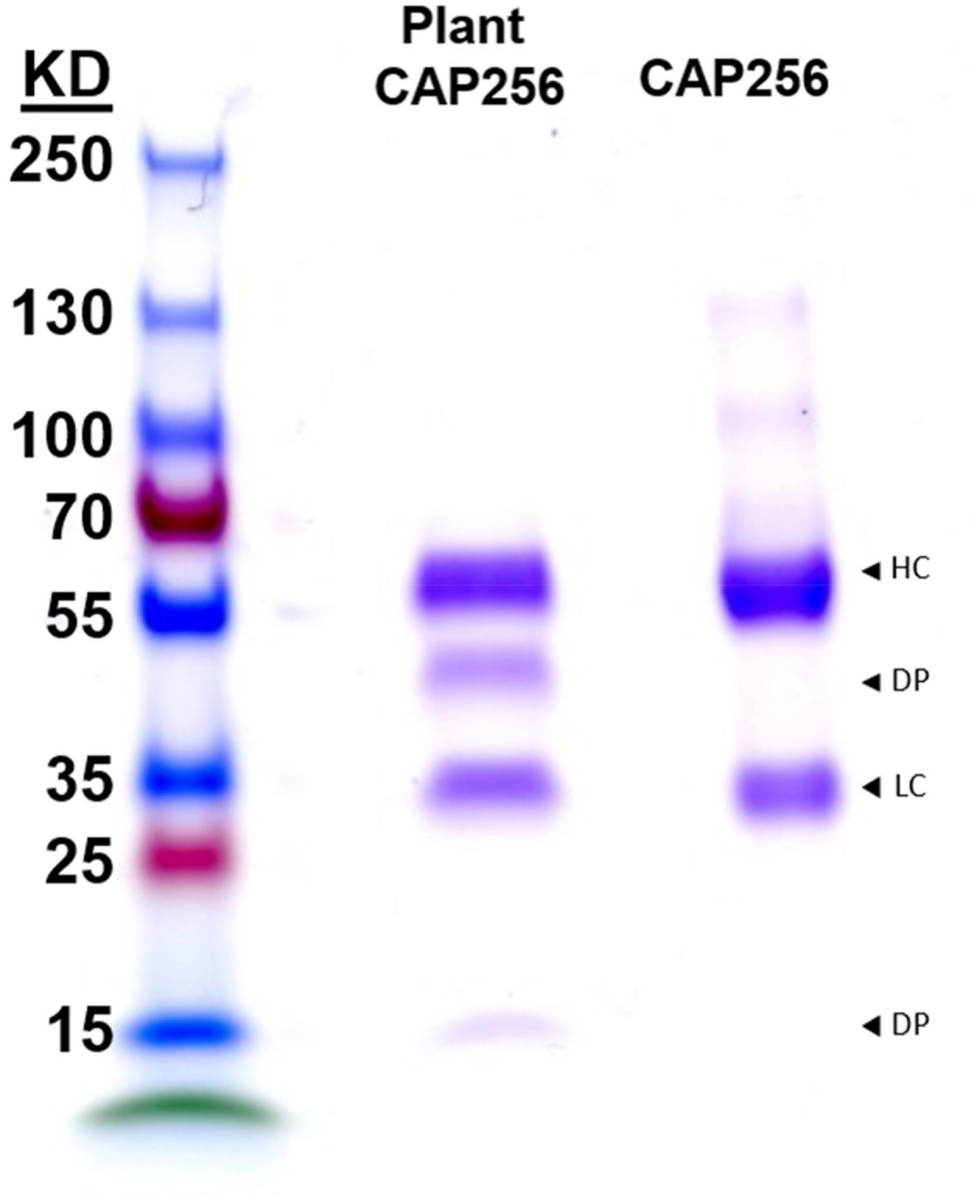
SDS-PAGE analysis of the plant CAP256-VRC26.25 mAbs produced in *N. benthamiana* (*Δ*XTFT) and Control produced in Mammalian cells. HC, Heavy chain; LC, Light Chain; DP, degradation product.

Following the unexpected pharmacokinetic findings in the macaque study, further analysis with SDS-PAGE under reducing conditions was undertaken to examine the structural integrity of the plant-produced antibody in greater detail. These analyses revealed evidence of partial proteolytic nicking within the antibody preparation (Figure 2) as a plausible mechanistic hypothesis requiring further investigation.

**Figure 3:**
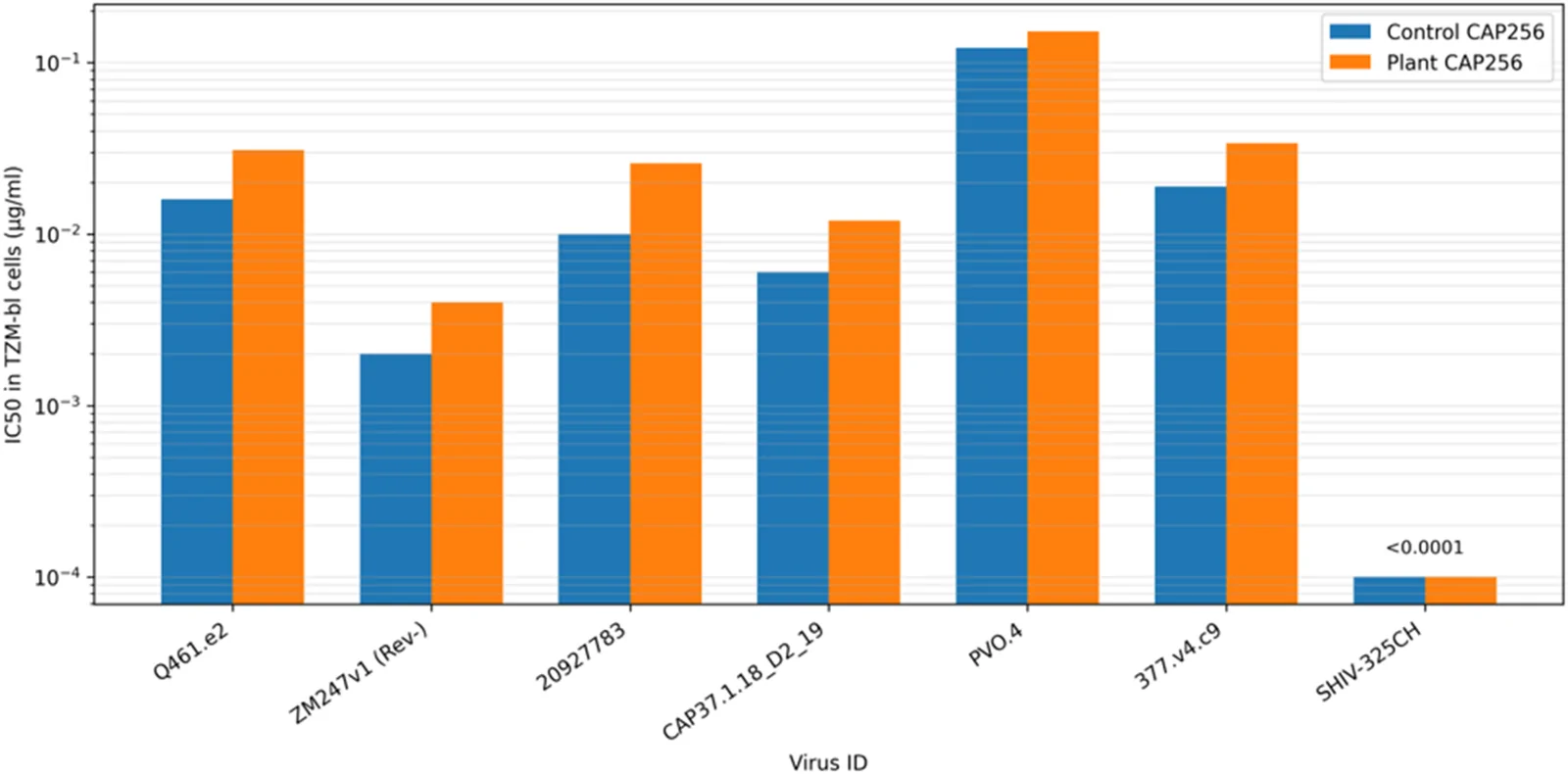
*In vitro* neutralization activity of CAP256-VRC26.25 preparations prior to *in vivo* evaluation. IC50 values measured in TZM-bl cells are shown for control CAP256-VRC26.25 and plant-produced CAP256-VRC26.25 across a panel of HIV/SHIV pseudoviruses. Lower IC50 values indicate greater neutralization potency. The plant-produced antibody retained measurable neutralizing activity across the panel prior to animal administration.

Proteolytic cleavage events affecting antibody molecules have been reported previously in plant-based expression systems and are attributed to endogenous plant proteases present during protein expression or extraction (52, 53). Such proteolytic modifications can occur without completely disrupting the overall quaternary structure of the antibody and therefore may not always be readily apparent in routine structural analyses. Importantly, *in vitro* neutralization assays conducted on the same batch used in the *in vivo* non-human primate study confirmed that the batch of plant-derived CAP256-VRC26.25 retained the expected neutralizing activity against HIV pseudoviruses, supporting the conclusion that reduced *in vivo* efficacy was not attributable to complete loss of antigen-binding or neutralization function prior to administration (Table 1, Figure 3).

**Table 1:** *In vitro* neutralization analysis of the CAP256-VRC26.25 batch administered to macaques.

	Titer in TZM-bl cells (µg/mL)			
	Control CAP256		Plant CAP256	
Virus ID	IC50	IC80	IC50	IC80
Q461.e2	0.016	0.564	0.031	1.044
ZM247v1 (Rev-)	0.002	0.028	0.004	0.062
20927783	0.010	0.869	0.026	2.724
CAP37.1.18_D2_19	0.006	0.036	0.012	0.077
PVO.4	0.122	0.983	0.152	2.121
377.v4.c9	0.019	2.165	0.034	1.353
SHIV-325CH	<0.0001	0.007	<0.0001	0.008
MuLV	>25	>25	20.582	>25

This finding indicated that the antigen-binding domains of the antibody remained functionally intact. However, partial proteolytic nicking affecting regions of the antibody outside the antigen-binding domain may nevertheless influence the stability or pharmacokinetic behavior of the molecule *in vivo* (54, 55). In addition, structural perturbations affecting the Fc region, for example, could alter interactions with Fc receptors that contribute to IgG stability and recycling in circulation (56).

Collectively, these observations suggest that proteolytic nicking of plant-derived CAP256-VRC26.25 may have been one of the contributing factors towards the reduced *in vivo* stability and rapid clearance observed in the non-human primate study. We previously found potential cleavage sites between amino acid positions 397 and 401 (50). Residues, which are responsible for FcRn binding are located between the CH2 and CH3 regions of the constant regions of the heavy chain (56, 57). Although the antibody retained neutralizing activity *in vitro*, the structural modifications detected in the plant-produced preparation likely affected its pharmacokinetic behavior *in vivo*. These findings highlight the importance of controlling host-derived proteolytic activity when producing complex antibody therapeutics in plant expression systems, a factor which is currently being further investigated in our ongoing work.

## 4 Engineering strategies informed by the stability limitations of plant-produced CAP256-VRC26.25

The analytical findings described above suggested that proteolytic nicking of plant-produced CAP256-VRC26.25 may have contributed towards the reduced *in vivo* stability and rapid clearance observed in the non-human primate study. Endogenous proteases present in plant tissues are known to affect the stability of recombinant proteins during expression and extraction, and proteolytic degradation has previously been reported as a challenge in plant-based antibody production (52, 53). Addressing this limitation is therefore an important step toward optimizing plant expression systems for the production of therapeutic monoclonal antibodies. The relationship between the present study and our previously published CAP256-VRC26.25 plant-expression work is important to clarify. Singh et al. (26) first reported plant-based production of CAP256-VRC26.25 with engineered post-translational modifications and demonstrated that the plant-produced antibody retained *in vitro* neutralising activity, while also identifying evidence of proteolytic cleavage. The non-human primate study reported here was initiated using this earlier-generation plant-produced antibody preparation to determine whether preserved *in vitro* neutralisation activity translated into *in vivo* pharmacokinetic exposure and protection. The study by Singh et al. (50) addressed the proteolytic susceptibility observed in the earlier material by demonstrating that transient CRISPR–Cas9-mediated suppression of endogenous *Nicotiana benthamiana* proteases could improve resistance to cleavage while retaining *in vitro* neutralisation activity. Thus, the present manuscript represents the *in vivo* translational evaluation of the earlier-generation plant-produced CAP256-VRC26.25 preparation, whereas the *in vivo* evaluation of improved cleavage-resistant candidates remains an important next step, yet to be conducted.

The present *in vivo* findings, together with the analytical observations from this currently reported work and earlier plant-expression studies, therefore served as an important translational trigger for subsequent and ongoing host- and antibody-engineering efforts and *in vivo* evaluation rather than representing the final optimized form of the platform. Recent advances in plant genome engineering provide opportunities to mitigate these challenges in iterative optimization cycles. In previous work, we demonstrated that transient CRISPR–Cas9-mediated editing can suppress specific endogenous proteases in *Nicotiana benthamiana*, thereby improving the stability of recombinant CAP256-VRC26 antibodies expressed in this system (50). This approach enables rapid modification of the plant host during transient expression and represents a flexible strategy for improving the quality of plant-produced biologics without requiring permanent genome modification of production lines. Host engineering approaches of this kind provide an important tool for addressing plant-specific degradation pathways that can affect the integrity of recombinant proteins.

In parallel, antibody sequence optimization represents an important complementary strategy for improving the manufacturability and stability of therapeutic antibodies. Engineering approaches have been applied to several HIV broadly neutralizing antibodies to enhance their pharmacokinetic properties and production characteristics. For example, Zhang et al. (58) reported the engineering of CAP256-VRC26V2LS variants produced in mammalian cell culture to improve manufacturability and extend antibody half-life while preserving neutralization potency (58). Importantly, their work demonstrated that certain manufacturability liabilities associated with the CAP256-VRC26 antibody lineage were conserved across multiple expression platforms, suggesting that intrinsic sequence features of the antibody can influence stability, expression efficiency, and downstream processing irrespective of the production host. Additional evidence supporting the presence of sequence-encoded stability liabilities within the CAP256-VRC26 lineage has been reported by Gollapudi et al. (59), who identified heavy-chain clipping events during manufacturing development of CAP256-VRC26LS antibodies produced in conventional expression systems (59). Their analytical characterization demonstrated that proteolytic cleavage occurred at defined sites within the antibody heavy chain, highlighting how intrinsic sequence features of the molecule can influence stability during production and processing. These observations reinforce the importance of considering both antibody sequence liabilities and host-specific factors when optimizing manufacturing strategies for broadly neutralizing antibodies.

Guided by the findings of the present study, together with insights from Singh et al., Zhang et al., and the analytical work of Gollapudi et al., ongoing work is focused on engineering CAP256-VRC26.25 variants in which predicted protease-sensitive sites are modified to reduce susceptibility to proteolytic cleavage during plant expression (50, 58, 59). These sequence modifications are designed to preserve antigen-binding activity while improving the structural stability of the antibody when produced in plant systems. The engineering pathway that has emerged from these findings is therefore iterative: host-directed approaches aim to reduce endogenous plant protease activity during transient expression, while antibody-directed approaches aim to modify predicted protease-sensitive sites within CAP256-VRC26.25 without compromising neutralization potency or structural integrity. Improved candidates will require comparative analytical characterization and pharmacokinetic evaluation to determine whether these interventions enhance *in vivo* durability. In this way, the current study defines the key translational constraints that subsequent optimization efforts are designed to address. By integrating insights from *in vivo* evaluation, structural analysis, host genome engineering, and antibody sequence optimization, it may be possible to develop plant-based production systems capable of generating stable, functional antibodies suitable for translational applications, including prevention of pediatric HIV infection.

## 5 Translational implications for monoclonal antibody development and access for children

Monoclonal antibodies are emerging as an important class of preventive interventions for infectious diseases affecting infants and children. In the context of HIV, broadly neutralizing antibodies have demonstrated the ability to prevent infection in non-human primate models and are currently being evaluated in clinical studies for prevention of HIV acquisition and vertical transmission (37, 60). Their high potency and long half-life make them particularly attractive candidates for protecting vulnerable populations such as infants and adolescent girls and young women. However, along with clinical efficacy, the global impact of monoclonal antibody-based prevention strategies will ultimately also depend on the ability to manufacture them at scale and at a cost that enables broad access. Manufacturing innovation has therefore been identified as a critical determinant of the affordability and global availability of antibody therapeutics (23).

The development of bnAbs for pediatric and global health applications therefore requires careful consideration of manufacturability and production platforms early in the development pathway. Conventional mammalian cell-based manufacturing remains the dominant platform for therapeutic antibodies but is associated with high infrastructure costs and complex bioprocessing requirements that can limit production capacity and affordability. Emerging manufacturing approaches, including continuous manufacturing, modular platforms and alternative expression systems, are increasingly being explored as strategies to expand global antibody supply and reduce production costs (23). Alternative production systems, including yeast (61) and plant-based expression platforms (43), have been proposed as potential approaches to reducing manufacturing costs and enabling more geographically distributed production of biologics.

The descriptive findings reported in this initial pilot translational evaluation study highlight both the potential and the challenges associated with alternative antibody production platforms. While limited in scope, this initial evaluation of plant-produced CAP256-VRC26.25 in a non-human primate challenge model provides a useful case study of how early *in vivo* assessment can reveal manufacturability and stability constraints that may not be fully apparent from *in vitro* potency assays alone. While plant expression systems offer attractive advantages in terms of relatively facile scalability, upstream costs and rapid production, the present results illustrate how host-specific factors, such as endogenous proteases, can influence the stability and pharmacokinetic behavior of complex antibody molecules. These properties are particularly important for antibodies intended for pediatric use, where maintaining adequate circulating antibody levels is critical for durable protection in infants and young children who may have limited dosing options and distinct pharmacokinetic profiles (62).

At the same time, evidence from previous studies indicates that certain manufacturability liabilities associated with CAP256-VRC26, and broadly neutralizing antibodies more generally, may be intrinsic to the antibody sequence itself and can manifest across production systems (56, 57). Studies of antibody developability have shown that structural liabilities and partial proteolytic cleavage affecting regions outside the antigen-binding domain can alter antibody stability, Fc-mediated functionality, and pharmacokinetic behavior without necessarily abolishing antigen recognition (52, 53). Recent analytical investigations of CAP256-VRC26 lineage antibodies produced in mammalian systems similarly identified heavy-chain clipping and sequence-associated stability liabilities during manufacturing development (57). Together, these observations emphasize the need for integrated approaches that combine antibody engineering, host optimization, and rigorous analytical characterization when developing monoclonal antibodies for translational applications. They have also informed ongoing work aimed at more comprehensive analytical characterization and iterative engineering of both the antibody sequence and plant expression host to better understand and mitigate the factors contributing to the observed pharmacokinetic behavior.

Importantly, the ability to identify such manufacturability challenges during early-stage development can help guide the design of more robust antibody candidates and production processes. Rather than representing a fully optimized plant-produced bNAb candidate, the present study identifies key stability and pharmacokinetic constraints that subsequent engineering efforts are designed to address. Iterative strategies combining host protease suppression, antibody sequence optimization, and rigorous analytical comparability assessment provide a rational framework for improving the stability and *in vivo* durability of plant-derived antibodies while preserving neutralization potency. For broadly neutralizing antibodies intended for global health applications, including prevention of mother-to-child transmission and protection of infants or adolescent girls at risk of HIV acquisition, such approaches may ultimately support the development of production processes capable of generating antibody candidates at greater scale and reduced cost. Overall, these findings illustrate how translational evaluation across manufacturing platforms can inform both biologic design and production strategy, and may contribute to the development of monoclonal antibody interventions that are more feasible for large-scale pediatric use. Future non-human primate pharmacokinetic and challenge studies of plant-produced CAP24]56-VRC26 will be required to determine whether the improved cleavage resistance achieved through host and antibody engineering translates into enhanced *in vivo* durability and protective activity.

## Data Availability

The original contributions presented in the study are included in the article/Supplementary Material, further inquiries can be directed to the corresponding author/s.

## Supplementary material

Supplementary Figure S1 **(A)** SDS,PAGE analysis of the eluates from the SEC purification of the CAP256-VRC26.25 mAbs produced in *N. benthamiana* (ΔXTFT). SDS-PAGE analysis of the Protein A and SEC purification of the CAP256-VRC26.25 mAbs. Analysis was done under non-reducing conditions. M represents the molecular weight marker (PageRuler™ Plus Prestained Protein Ladder). **(B)** Overlaid chromatogram of the HPLC–SEC analysis of the CAP256-VRC26 mAbs HPLC-SEC chromatogram of the CAP256-VRC26.25 mAbs analysed under non-reducing conditions. Chromatogram of CAP256-VRC26.25 mAbs produced in HEK293 (blue) and in *N. benthamiana* (red). Under native (non-reducing) conditions, both preparations showed the same retention time.

Supplemental Table S1 Summary of constructs and strains used in this study.

Supplemental Table S2 Sequencing of CAP256-VRC26.25 plant-expression studies and their respective translational contributions.
