# Supplementary material for: Translational insights into manufacturability and stability of broadly neutralizing antibodies for pediatric HIV prevention: lessons from plant-produced CAP256-VRC26.25
Source: Front Pediatr. 2026 Aug 10;14:1837801. doi: 10.3389/fped.2026.1837801 (PMC13500764; doi:10.3389/fped.2026.1837801)
Supplement: Supplementary file 1 [file Supplementaryfile1.docx]

## Supplemental: Materials and Methods

### Expression Vector Construction and Plant Growth Conditions

Briefly, the variable region sequences were sourced from previously published (35, 60) and GenBank (KT371100.1 (protein ID: ALG00386.1)) and KT371101.1 (protein ID: ALG00387.1) (63, 64). The heavy and light chain variable regions for the CAP256-VRC26.25 were fused to murine signal peptides (SPs) and human IgG1 lambda and gamma constant regions. Genes were inserted into pICH31180 and pICH21161 vectors with non-competing Potato Virus X (PVX) and Tobacco Mosaic Virus (TMV) MagnICON vector backbones sourced from ICON Genetics and Nomad Bioscience (DE) (65). The constructs prepared for this study are summarized in (65). The construct for hTPST1 was a gift from Professor Herta Steinkellner and Professor Lukas Mach (Department of Applied Genetics and Cell Biology, University of Natural Resources and Life Sciences, Vienna, Austria). The sequence encoding hTPST1 was inserted into the binary vector pPT2 (66). *N. benthamiana* plants were grown and prepared at the growth room facility, first floor of building 20, room N142 at (CSIR, Pretoria, South Africa), at 25℃ with a 16-hour light, 8-hour dark photoperiod, for 4-5 weeks before agroinfiltration.

### Agrobacterium transformation

The transformation of the CAP256-VRC26.25 constructs and hTPST1 constructs into competent *A. tumefaciens* LBA4404 (Invitrogen, USA) was conducted as previously described (26). These transformed strains are referred to as *A. tumefaciens* strains, unless otherwise stated. *A. tumefaciens* strains were transformed by electroporation using a Gene Pulser electroporator (Biorad, USA) with 100 ng of DNA using a 1 mm electroporation cuvette (Sigma-Aldrich, USA) at 1.44 kV, 200 Ω, and 25 uF. The transformed *A. tumefaciens* strains were immediately recovered in 1 mL Luria broth (LB) (10 g.L^-1^ Tryptone (Oxoid Ltd., UK), 10 g.L^-1^ Sodium chloride (Merck KGaA, DE), 5 g.L^-1^, Yeast extract (Merck KGaA, DE), pH 7.4) and incubated at 30°C for three hours at 200 rpm agitation. The transformed *A. tumefaciens* strains were plated on to LB agar (10 g.L^-1^ Tryptone (Oxoid Ltd., UK), 10 g.L^-1^ Sodium chloride (Merck KGaA, DE), 5 g.L^-1^ Yeast extract (Merck KGaA, DE), 15 g.L^-1^ Bacteriological agar (Oxoid Ltd., UK), pH 7.4) plates supplemented with 50 µg.mL^-1^ Kanamycin (Sigma-Aldrich, USA) and 25 µg.mL^-1^ Rifampicin (Sigma-Aldrich, USA) and were grown for 48 hours at 28°C. Colony selection for each construct was done and inoculated into 10 mL of LB supplemented with the same antibiotics. The cultures were further incubated at 28°C and 200 rpm agitation in an MRC refrigerated orbital shaker incubator (600x480 mm plate) (MRC labs, UK) till an OD600 of 0.5 - 0.6 was reached. Thereafter, the cultures were cooled at 4°C for 30 min and harvested at 4000*g* for 15 min at 4°C using an Eppendorf ® 5810R centrifuge (Eppendorf, DE), with the supernatants discarded thereafter. The pelleted cells were thereafter re-suspended in 10 mL LB/ 20 % glycerol (Merck KGaA, DE) and aliquoted into 1 mL aliquots and stored at -80°C.

### Agroinfiltration of ΔXF *N. benthamiana* plants

CAP256-VRC26.25 was expressed using the *Agrobacterium*-mediated transient expression system (67). Briefly, LB containing above mentioned antibiotics were inoculated with *A. tumefaciens* with the constructs encoding CAP256-VRC26.25 light chain (LC), CAP256-VRC26.25 heavy chain (HC) and hTPST1 and were inoculated in a 1:200 with the respective transformed *A. tumefaciens* glycerol stocks and incubated in a MRC refrigerated orbital shaker incubator (600x480 mm plate) for 48 hours at 28 ^o^C and 200 rpm agitation. *Agrobacterium tumefaciens* cells were centrifugally harvested at 4000*g* for 20 min at 4^°^C using an Eppendorf® 5810R (Eppendorf, DE) and the supernatants discarded thereafter. Pelleted *Agrobacterium* was resuspended in 20 mL infiltration buffer (10 mM 2-(N-morpholino)ethanesulfonic acid (MES) (Sigma-Aldrich, USA), 10 mM MgCl_2_ (Sigma-Aldrich, USA), pH 5.5). An OD_600_ of 0.01 for each CAP256-VRC26.25 chain was used, with an OD_600_ of 0.8 used for hTPST1. Syringe agroinfiltration was conducted as previously described (67). *Nicotiana benthamiana* (ΔXTFT) plants were grown in a plant growth room where conditions were maintained at 25^o^C constant temperature and a 16/8 hour light/dark cycle. During the light cycle, the photosynthesis photon flux density was 600 ± 50 µmol. m^-2^.s^-1^. Six weeks old *N. benthamiana* (ΔXTFT) plants were selected for syringe agroinfiltration. The waxy cuticle on the abaxial side of the *N. benthamiana* (ΔXTFT) leaf was lightly pricked using a sterile needle. A 1 mL syringe was used to inject the agroinfiltration suspensions in between the waxy cuticle of the leaf and the epidermal layer through the prick taking care to avoid the midrib of the leaf. CAP256-VRC26.25 expression was allowed to accumulate up to eight days post infiltration (dpi).

### Protein extraction, Quantification & Purification

Six hundred grams of leaf material was harvested at eight dpi. Leaf material was homogenised using a Matstone 6-in-one juice extractor (Matstone, PH). During the homogenisation process, phosphate buffered saline (PBS) (15 mM KH_2_PO_4_ (Merck KGaA, DE), 81 mM NaHPO_4_ (Merck KGaA, DE), 27 mM KCl (Merck KGaA, DE) and 140 mM NaCl (Merck KGaA, DE), pH 7.4) was added to the leaf material in a ratio of 1:2 (w/v). Leaf extracts were clarified using a Beckman Coulter Avanti J-26XPI (USA) at 8000*g* for 30 min at 4^o^C. The centrifugation step was repeated four times. CAP256-VRC26.25 was purified using a 5 mL HiTrap® Protein A High Performance column (Cytiva LifeSciences, USA) coupled to an Akta Avant 150 (Cytiva Life Sciences, USA). The HiTrap® Protein A High Performance column was equilibrated with 50 mM Tris, pH 7.4 at 2 ml/min for 6 column volumes (CVs). Clarified leaf extracts were loaded on the column at 2 ml/min. The column was thereafter washed at 2 ml/min using 50 mM Tris, pH 7.4 for 30 CVs. The bound CAP256-VRC26.25 was eluted of the column isocratically using a 2.5% acetic acid elution buffer. The protein A purified N. benthamiana (ΔXTFT)-produced CAP256-VRC26 mAbs was further Purified using size exclusion chromatography (SEC) using a Sephadex 200 pg 16/600 (Cytiva LifeSciences, USA) coupled to an Akta Avant 150 (Cytiva LifeSciences, USA). A mobile phase of 50 mM Na_2_HPO_4_, 150 mM NaCl, 200 mM L-Arginine pH 7.4 was used, with the Sephadex 200 pg 16/600 column being equilibrated at 1 ml/min for 2 CVs. 5 mL of mAb sample was loaded onto the Sephadex 200 pg 16/600 column at 1 ml/min from a 5 mL capillary loop. mAbs were eluted at 1 ml/min and collected in 1 mL fraction volumes in a 96 well fraction collector. Purified samples were assessed electrophoretically using Bolt™ 4-12% Bis-Tris Plus Gels (Invitrogen, USA), as per the manufacturer’s instructions, as well as via Western blotting under reducing or non-reducing conditions. The concentration of the CAP256-VRC26.25 samples were measured against a standard curve which was prepared using the Quick Start™ Bovine γ-globulin standard set (Bio-Rad, USA). Briefly, the bovine γ-globulin standard curve was prepared by the addition of 5 µL of each standard (0.125 – 1.5 mg/mL) into 245 µL Quick Start™ Bradford 1x Dye Reagent. This was done in triplicate. The concentration of the CAP256-VRC26.25 samples were diluted with 50 mM Na_2_HPO_4_, 150 mM NaCl, 200 mM L-Arginine pH 7.4, in 1:2, 1:4 and 1:8 dilution. 5 µL *of each* CAP256-VRC26.25 dilution was added into 245 µL Quick Start™ Bradford 1x Dye Reagent. This was done in triplicate. 200 µL of the standard and CAP256-VRC26.25 samples were added to a 96 well microtiter plate. The absorbance was read at 595 nm using a Hidex Sense Microplate Reader (Hidex, EE).

### Quantification of endotoxins

The concentration of endotoxins was measured using the Pierce™ LAL Chromogenic Endotoxin Quantitation kit as per the manufacturer’s guidelines. Briefly, *E. coli* endotoxin standards were prepared from the dilution of 15 EU stock solution using endotoxin-free water (EFW). A standard curve of 0.1 – 1.5 EU/mL was prepared. The lyophilized amebocyte lysate was reconstituted immediately before use with 1.7 mL of EFW and gently swirled to dissolve the powder. The 96 well plate used in the assay was pre-equilibrated to 37^o^C using a plate heater. 50 μL of endotoxin standard dilutions, blank, and samples were added per well in triplicates. While keeping the plate at 37^o^C, 50 μL of the reconstituted amebocyte lysate reagent was added to each well and removed from the 37^o^C plate heater and mixed by gentle tapping of the plate. The plate was returned to the 37^o^C plate heater and incubated for 1 min. The chromogenic substrate was reconstituted in a 3.4 mL of EFW and mixed by gently tilting and swirling the vial. The substrate was thereafter pre-warmed to 37^o^C for 5 min before use. 100 μL of the pre-warmed reconstituted chromogenic substrate was added per a well. The plate was removed from the plate heaters and mix gently by tapping 10 times to facilitate mixing. The plate was returned to the plate heater and incubated for a further 6 min at 37^o^C, after which stop solution (25% acetic acid) was added to each well. The plate was thereafter removed from the plate heater and gently mixed by tapping the plate 10 times. The absorbance was read at 405 nm using a Hidex Sense Microplate Reader (Hidex, EE). A standard curve was prepared by plotting the blank-corrected absorbance for each standard on the y-axis vs. the corresponding endotoxin concentration in EU/mL on the x-axis.

### *In vitro* functional characterisation studies of the CAP256-VRC26.25 mAbs

Neutralisation efficacy of the *N. benthamiana* (ΔXTFT)-produced CAP256-VRC26.25 was assessed pre-shipment. The anti-HIV-1 activity was screened using the TZM-bl neutralisation assay as previously described (68). The neutralization efficacy of *N. benthamiana* (ΔXTFT)-produced CAP256-VRC26.25 was assessed against CAP256-VRC26.25 which had been produced in HEK293 cells. A threefold dilution series of each antibody was prepared in Dulbecco’s Modified Eagle’s medium (DMEM) (Sigma-Aldrich Co., USA) with 10 % Fetal Bovine Serum (FBS) (Sigma-Aldrich Co., USA) (growth media), with 100 µL in a 96-well plate in duplicates. Two hundredths the 50 % tissue culture infective dose (TCID_50_) of the pseudovirus in 50 μL of growth medium was added to each antibody containing well, followed by incubation for one hour at 37°C. To each well, 100 μL of TZM-bl cells at a concentration of 1 × 10^4^ cells/100 µL containing 37.5 μg.mL^-1^ of DEAE dextran was added and cultured at 37°C for 48 hours. The Bright Glo Reagent (Promega, USA) was used as per the manufacturer’s guidelines. Luminescence measurements were taken using an Infinite F500 plate reader (Tecan, Austria). The evaluation of the inhibition of HIV-1 infection was done by measurement of the activity of the firefly luciferase. Titres were calculated as the inhibitory concentration that causes 50% reduction (IC50) of the relative light unit (RLU) compared to the virus control (wells with no CAP256-VRC26 mAbs) after the subtraction of the background (wells without both the virus and the CAP256-VRC26 mAbs).

### Size-exclusion chromatography

Size-exclusion chromatography was performed on an Agilent 1100 series HPLC system (Agilent Technologies Inc., DE) which consisted of a binary pump, degasser, well-plate autosampler (WPALS), autosampler thermostat (ALS Thermo), column thermostat compartment and a diode-array detector (DAD). All data analysis was performed using Chemstation chromatography data management software, revision B.04.02 (Agilent Technologies Inc., DE). The analytical column used was an AdvanceBio SEC 300 Å 2.7 um, 7.8 x 300 mm column (Agilent Technologies Inc., DE). The mobile phase contained 150 mM Na_2_HPO_4_ (Merck KGaA, DE), pH 7. The column was conditioned for 1h with mobile phase before the injection of samples. CAP256-VRC26.25 mAbs were eluted isocratically at a flow rate of 0.35 mL/min for 20 min and the column effluent was monitored at 220 nm. All data was collected at 30 °C.

### Non-human primate challenge study

The *in vivo* evaluation of CAP256-VRC26.25 was conducted in cynomolgus macaques (Macaca fascicularis). Nine animals were assigned to three groups (n = 3 per group) and received a single intravenous infusion of either plant-produced CAP256-VRC26.25, mammalian cell-derived CAP256-VRC26.25 (control), or vehicle (sham control) at a dose of 1 mg/kg on Day −3 relative to viral challenge. Three days following antibody administration, all animals were challenged via the intrarectal route with SHIV-325cH. All animal procedures were performed in accordance with institutional guidelines and approved animal care and use protocols. While limited, the group sizes used are broadly consistent with exploratory study designs and challenge models based on established non-human primate protocols for evaluating the prophylactic efficacy of HIV broadly neutralising antibodies, as previously described by Barouch and colleagues (37, 49). Serum samples were collected longitudinally following antibody administration to assess pharmacokinetics. Antibody concentrations were measured using antigen-specific binding assays, and pharmacokinetic parameters were derived from serum concentration-time profiles, as previously described for bnAb studies. Following SHIV-325cH challenge, plasma viral loads were quantified, and infection outcomes and viral kinetics were monitored longitudinally (37, 49).

## Supplemental Figures

**Supplementary Figure 1. (A) SDS-PAGE analysis of the eluates from the SEC purification of the CAP256-VRC26.25 mAbs produced in *N. benthamiana* (ΔXTFT).** SDS-PAGE analysis of the Protein A and SEC purification of the CAP256-VRC26.25 mAbs. Analysis was done under non-reducing conditions. M represents the molecular weight marker (PageRuler™ Plus Prestained Protein Ladder). **(B) Overlaid chromatogram of the HPLC-SEC analysis of the CAP256-VRC26 mAbs** HPLC-SEC chromatogram of the CAP256-VRC26.25 mAbs analysed under non-reducing conditions. Chromatogram of CAP256-VRC26.25 mAbs produced in HEK293 (blue) and in *N. benthamiana* (red). Under native (non-reducing) conditions, both preparations showed the same retention time.

## Supplemental Tables

| Construct Full name | Labelling Convention | Microorganism transformed | Strain | Vector antibiotic selection | Microorganism antibiotic selection |
| --- | --- | --- | --- | --- | --- |
| Originator 25 HC Magnicon |  | *A. tumefaciens* | LBA4404 | Kanamycin | Rifampicin, Streptomycin |
| Originator 25 LC Magnicon |  | *A. tumefaciens* | LBA4404 |  | Rifampicin, Streptomycin |
| pPT2-hTPST1 |  | *A. tumefaciens* | GV3101::pMP90 |  | Rifampicin, Gentamicin |

**Supplemental Table 1**. Summary of constructs and strains used in this study.

**Supplemental Table 2**. Sequencing of CAP256-VRC26.25 plant-expression studies and their respective translational contributions.

The table summarizes the relationship between the previously published plant-production and host-engineering studies and the present non-human primate evaluation. Singh *et al*. (2020) established plant-based production of CAP256-VRC26.25 and demonstrated retained *in vitro* neutralization activity despite evidence of proteolytic cleavage. The present study evaluated the earlier-generation plant-produced antibody preparation *in vivo* and demonstrated poor pharmacokinetics and lack of protection despite retained *in vitro* neutralization activity. Singh *et al*. (2022) demonstrated improved resistance to proteolytic cleavage through transient CRISPR–Cas9-mediated suppression of endogenous *Nicotiana benthamiana* proteases, while preserving *in vitro* neutralization activity.

| Study | Cleavage addressed? | In vitro neutralisation assessed? | In vivo activity assessed? | Main contribution |
| --- | --- | --- | --- | --- |
| Singh et al. 2020 | No | Yes | No | Established plant production of CAP256-VRC26.25 and identified proteolytic cleavage despite retained *in vitro* activity. |
| Present study | No | Yes | Yes | Demonstrated that earlier-generation plant-produced CAP256-VRC26.25 had poor *in vivo* pharmacokinetics and failed to protect despite retained *in vitro* activity. |
| Singh et al. 2022 | Yes | Yes | No | Demonstrated improved cleavage resistance through transient CRISPR–Cas9 protease suppression while retaining *in vitro* activity. |
