# Supplementary material for: Translational insights into manufacturability and stability of broadly neutralizing antibodies for pediatric HIV prevention: lessons from plant-produced CAP256-VRC26.25
Source: Front Pediatr. 2026 Aug 10;14:1837801. doi: 10.3389/fped.2026.1837801 (PMC13500764; doi:10.3389/fped.2026.1837801)
Supplement: Supplementary file 2 [file Supplementaryfile2.docx]

**References**

1. UNAIDS. UNAIDS. Global HIV & AIDS statistics — fact sheet (2026). Available online at: https://www.unaids.org/en/resources/fact-sheet (accessed [Jul 31, 2026])

2. Mutabazi JC, Zarowsky C, Trottier H. The impact of programs for prevention of mother-to-child transmission of HIV on health care services and systems in sub-Saharan Africa-A review. *Public Health Rev.* (2017) 38(1):28. doi: 10.1186/s40985-017-0072-5

3. Organization WH. Guidance on global scale-up of the prevention of mother-to-child transmission of HIV (2007).

4. Abuogi LL, Humphrey JM, Mpody C, Yotebieng M, Murnane PM, Clouse K, et al. Achieving UNAIDS 90-90-90 targets for pregnant and postpartum women in sub-Saharan Africa: progress, gaps and research needs. *J Virus Erad.* (2018) 4:33–9. doi: 10.1016/S2055-6640(20)30343-5

5. HIV/AIDS JUNPo. HIV prevention 2025–road map: Getting on Track to end AIDS as a Public Health Threat by 2030. Geneva, Switzerland: UNAIDS (2022).

6. Millett GA, Crowley JS, Koh H, Valdiserri RO, Frieden T, Dieffenbach CW, et al. A way forward: the national HIV/AIDS strategy and reducing HIV incidence in the United States. *JAIDS J Acquir Immune Defic Syndr.* (2010) 55:S144–7. doi: 10.1097/QAI.0b013e3181fbcb04

7. Kumah E, Boakye DS, Boateng R, Agyei E. Advancing the global fight against HIV/AIDS: strategies, barriers, and the road to eradication. *Ann Glob Health.* (2023) 89(1):83. doi: 10.5334/aogh.4277

8. Burton DR, Hangartner L. Broadly neutralizing antibodies to HIV and their role in vaccine design. *Annu Rev Immunol.* (2016) 34(1):635–59. doi: 10.1146/annurevimmunol-041015-055515

9. Julg B, Barouch D, editors. Broadly neutralizing antibodies for HIV-1 prevention and therapy. *Semin Immunol.* (2021) 51:101475. doi: 10.1016/j.smim.2021.101475

10. Huang Y, Naidoo L, Zhang L, Carpp LN, Rudnicki E, Randhawa A, et al. Pharmacokinetics and predicted neutralisation coverage of VRC01 in HIV-uninfected participants of the antibody mediated prevention (AMP) trials. *EBioMedicine.* (2021) 64:103203. doi: 10.1016/j.ebiom.2020.103203

11. Miner MD, Corey L, Montefiori D. Broadly neutralizing monoclonal antibodies for HIV prevention. *J Int AIDS Soc.* (2021) 24:e25829. doi: 10.1002/jia2.25829

12. Zalevsky J, Chamberlain AK, Horton HM, Karki S, Leung IWL, Sproule TJ, et al. Enhanced antibody half-life improves in vivo activity. *Nat Biotechnol.* (2010) 28(2):157–9. doi: 10.1038/nbt.1601

13. Ko S, Jo M, Jung ST. Recent achievements and challenges in prolonging the serum half-lives of therapeutic IgG antibodies through fc engineering: a. Ko et al. *BioDrugs.* (2021) 35(2):147–57. doi: 10.1007/s40259-021-00471-0

14. Van de Perre P, Scarlatti G, Moore PL, Molès J-P, Nagot N, Tylleskär T, et al. Preventing breast milk HIV transmission using broadly neutralizing monoclonal antibodies: one size does not fit all. *Immun Inflamm Dis.* (2024) 12(3):e1216. doi: 10.1002/iid3.1216

15. Obeagu EI. HIV and maternal-fetal immunity: challenges and strategies for reducing vertical transmission-A narrative review. *Health Sci Rep.* (2025) 8(7):e71076. doi: 10.1002/hsr2.71076

16. Mangold JF, Goswami R, Nelson AN, Martinez DR, Fouda GG, Permar SR. Maternal intervention to prevent mother-to-child transmission of HIV: moving beyond antiretroviral therapy. *Pediatr Infect Dis J.* (2021) 40(5S):S5–S10. doi: 10.1097/INF.0000000000002774

17. Cunningham CK, McFarland EJ, Morrison RL, Capparelli EV, Safrit JT, Mofenson LM, et al. Safety, tolerability, and pharmacokinetics of the broadly neutralizing human immunodeficiency virus (HIV)-1 monoclonal antibody VRC01 in HIV-exposed newborn infants. *J Infect Dis.* (2020) 222(4):628–36. doi: 10.1093/infdis/jiz532

18. Tebas P. Future of bNAbs in HIV treatment. *Curr HIV/AIDS Rep.* (2025) 22(1):34. doi: 10.1007/s11904-025-00744-1

19. Walsh G. Biopharmaceutical benchmarks 2018. *Nat Biotechnol.* (2018) 36(12):1136–45. doi: 10.1038/nbt.4305

20. Kelley B, editor. Industrialization of mAb production technology: the bioprocessing industry at a crossroads. *MAbs.* (2009) 1:443–52. doi: 10.4161/mabs.1.5.9448

21. Plotkin S, Robinson JM, Cunningham G, Iqbal R, Larsen S. The complexity and cost of vaccine manufacturing–an overview. *Vaccine.* (2017) 35(33):4064–71. doi: 10.1016/j.vaccine.2017.06.003

22. Malhotra S, Cameron A-I, Gotham D, Burrone E, Gardner PJ, Loynachan C, et al. Novel approaches to enable equitable access to monoclonal antibodies in low- and middle-income countries. *PLOS Global Public Health.* (2024) 4(7):e0003418. doi: 10.1371/journal.pgph.0003418

23. Gardner PJ, Loynachan C, Tsekoa TL, Keir L, Yabannavar V, Sparrow E. Manufacturing innovation is essential for monoclonal antibody affordability and access. *Nat Rev Bioeng.* (2025) 3(6):438–40. doi: 10.1038/s44222-025-00301-w

24. Ranbhor R. Advancing monoclonal antibody manufacturing: process optimization, cost reduction strategies, and emerging technologies. *Biologics.* (2025) Volume 19:177–87. doi: 10.2147/BTT.S515078

25. Tsekoa TL, Singh AA, Buthelezi SG. Molecular farming for therapies and vaccines in Africa. *Curr Opin Biotechnol.* (2020) 61:89–95. doi: 10.1016/j.copbio.2019.11.005

26. Singh AA, Pooe O, Kwezi L, Lotter-Stark T, Stoychev SH, Alexandra K, et al. Plant-based production of highly potent anti-HIV antibodies with engineered posttranslational modifications. *Sci Rep.* (2020) 10(1):1–9. doi: 10.1038/s41598-020-63052-1

27. Shanmugaraj B, I Bulaon CJ, Phoolcharoen W. Plant molecular farming: a viable platform for recombinant biopharmaceutical production. *Plants.* (2020) 9(7):842. doi: 10.3390/plants9070842

28. Chen Q, Davis KR. The potential of plants as a system for the development and production of human biologics. *F1000Res.* (2016) 5:912. doi: 10.12688/f1000research.8010.1

29. Liu H, Timko MP. Improving protein quantity and quality—the next level of plant molecular farming. *Int J Mol Sci.* (2022) 23(3):1326. doi: 10.3390/ijms23031326

30. Akher SA, Wang KY, Hall K, Hunpatin OS, Shan M, Zhang Z, et al. Harnessing transient expression systems with plant viral vectors for the production of biopharmaceuticals in Nicotiana benthamiana. *Int J Mol Sci.* (2025) 26(12):5510. doi: 10.3390/ijms26125510

31. Chung YH, Church D, Koellhoffer EC, Osota E, Shukla S, Rybicki EP, et al. Integrating plant molecular farming and materials research for next-generation vaccines. *Nature Reviews Materials.* (2022) 7(5):372–88. doi: 10.1038/s41578-021-00399-5

32. Vo D-K, Trinh KTL. Molecular farming for immunization: current advances and future prospects in plant-produced vaccines. *Vaccines (Basel).* (2025) 13(2):191. doi: 10.3390/vaccines13020191

33. Gomord V, Sourrouille C, Fitchette A-C, Bardor M, Pagny S, Lerouge P, et al. Production and glycosylation of plant-made pharmaceuticals: the antibodies as a challenge. *Plant Biotechnol J.* (2004) 2(2):83–100. doi: 10.1111/j.1467-7652.2004.00062.x

34. Jadhav RR, Khare D. Green biotherapeutics: overcoming challenges in plant-based expression platforms. *Plant Biotechnol Rep.* (2024) 18(4):465–86. doi: 10.1007/s11816-024-00910-8

35. Doria-Rose NA, Schramm CA, Gorman J, Moore PL, Bhiman JN, DeKosky BJ, et al. Developmental pathway for potent V1V2-directed HIV-neutralizing antibodies. *Nature.* (2014) 509(7498):55–62. doi: 10.1038/nature13036

36. Julg B, Tartaglia LJ, Keele BF, Wagh K, Pegu A, Sok D, et al. Broadly neutralizing antibodies targeting the HIV-1 envelope V2 apex confer protection against a clade C SHIV challenge. *Sci Transl Med.* (2017) 9(406):eaal1321. doi: 10.1126/scitranslmed.aal1321

37. Barouch DH, Stephenson KE, Borducchi EN, Smith K, Stanley K, McNally AG, et al. Protective efficacy of a global HIV-1 mosaic vaccine against heterologous SHIV challenges in rhesus monkeys. *Cell.* (2013) 155(3):531–9. doi: 10.1016/j.cell.2013.09.061

38. Masuda K, Kubota M, Nakazawa Y, Iwama C, Watanabe K, Ishikawa N, et al. Establishment of a novel cell line, CHO-MK, derived from Chinese hamster ovary tissues for biologics manufacturing. *J Biosci Bioeng.* (2024) 137:471–9. doi: 10.1016/j.jbiosc.2024.02.005

39. Majumdar S, Desai R, Hans A, Dandekar P, Jain R. From efficiency to yield: exploring recent advances in CHO cell line development for monoclonal antibodies. *Mol Biotechnol.* (2025) 67(2):369–92. doi: 10.1007/s12033-024-01060-6

40. Saha D, Das PK, Veeranki VD. Developments in the production of recombinant monoclonal antibodies. *Biotechnol J.* (2025) 20(10):e70127. doi: 10.1002/biot.70127

41. Xu W-J, Lin Y, Mi C-L, Pang J-Y, Wang T-Y. Progress in fed-batch culture for recombinant protein production in CHO cells. *Appl Microbiol Biotechnol.* (2023) 107(4):1063–75. doi: 10.1007/s00253-022-12342-x

42. Rathore AS, Metya S, Sharma R, Nitika N. Challenges and developments in the manufacturing of high-concentration biotherapeutic monoclonal antibodies. *Adv Drug Delivery Rev.* (2026) 232:115812. doi: 10.1016/j.addr.2026.115812

43. Nandi S, Kwong AT, Holtz BR, Erwin RL, Marcel S, McDonald KA, editors. Techno-economic analysis of a transient plant-based platform for monoclonal antibody production. *MAbs.* (2016) 8:1456–66. doi: 10.1080/19420862.2016.1227901

44. Virdi V, Juarez P, Boudolf V, Depicker A. Recombinant IgA production for mucosal passive immunization, advancing beyond the hurdles. *Cell Mol Life Sci.* (2016) 73(3):535–45. doi: 10.1007/s00018-015-2074-0

45. Paul M, Reljic R, Klein K, Drake PMW, Van Dolleweerd C, Pabst M, et al., editors. Characterization of a plant-produced recombinant human secretory IgA with broad neutralizing activity against HIV. *MAbs.* (2014) 6:1585–97. doi: 10.4161/mabs.36336

46. Göritzer K, Strasser R, Ma JK-C. Stability engineering of recombinant secretory igA. *Int J Mol Sci.* (2024) 25(13):6856. doi: 10.3390/ijms25136856

47. Göritzer K, Melnik S, Schwestka J, Arcalis E, Drapal M, Fraser P, et al. Enhancing quality and yield of recombinant secretory IgA antibodies in Nicotiana benthamiana by endoplasmic reticulum engineering. *Plant Biotechnol J.* (2025) 23(4):1178–89. doi: 10.1111/pbi.14576

48. Gorman J, Chuang G-Y, Lai Y-T, Shen C-H, Boyington JC, Druz A, et al. Structure of super-potent antibody CAP256-VRC26.25 in complex with HIV-1 envelope reveals a combined mode of trimer-apex recognition. *Cell Rep.* (2020) 31(1):107488. doi: 10.1016/j.celrep.2020.03.052

49. Mahomed S, Garrett N, Capparelli EV, Osman F, Mkhize NN, Harkoo I, et al. Safety and pharmacokinetics of escalating doses of neutralising monoclonal antibody CAP256V2LS administered with and without VRC07-523LS in HIV-negative women in South Africa (CAPRISA 012B): a phase 1, dose-escalation, randomised controlled trial. *Lancet HIV.* (2023) 10(4):e230–43. doi: 10.1016/S2352-3018(23)00003-6

50. Singh AA, Pillay P, Naicker P, Alexandre K, Malatji K, Mach L, et al. Transient proteolysis reduction of Nicotiana benthamiana-produced CAP256 broadly neutralising antibodies using CRISPR/Cas9. *Front Plant Sci.* (2022) 13:953654. doi: 10.3389/fpls.2022.953654

51. Barouch DH, Whitney JB, Moldt B, Klein F, Oliveira TY, Liu J, et al. Therapeutic efficacy of potent neutralizing HIV-1-specific monoclonal antibodies in SHIV-infected rhesus monkeys. *Nature.* (2013) 503(7475):224–8. doi: 10.1038/nature12744

52. Benchabane M, Goulet C, Rivard D, Faye L, Gomord V, Michaud D. Preventing unintended proteolysis in plant protein biofactories. *Plant Biotechnol J.* (2008) 6(7):633–48. doi: 10.1111/j.1467-7652.2008.00344.x

53. Niemer M, Mehofer U, Torres Acosta JA, Verdianz M, Henkel T, Loos A, et al. The human anti-HIV antibodies 2F5, 2G12, and PG9 differ in their susceptibility to proteolytic degradation: down-regulation of endogenous serine and cysteine proteinase activities could improve antibody production in plant-based expression platforms. *Biotechnol J.* (2014) 9(4):493–500. doi: 10.1002/biot.201300207

54. Brezski RJ, Jordan RE, editors. *Cleavage of IgGs by proteases associated with invasive diseases: an evasion tactic against host immunity? MAbs.* (2010) 2:212–20. doi: 10.4161/mabs.2.3.11780

55. Brezski RJ, Vafa O, Petrone D, Tam SH, Powers G, Ryan MH, et al. Tumor-associated and microbial proteases compromise host IgG effector functions by a single cleavage proximal to the hinge. *Proc Natl Acad Sci USA.* (2009) 106(42):17864–9. doi: 10.1073/pnas.0904174106

56. Roopenian DC, Akilesh S. Fcrn: the neonatal fc receptor comes of age. *Nat Rev Immunol.* (2007) 7(9):715–25. doi: 10.1038/nri2155

57. Kim JK, Firan M, Radu CG, Kim CH, Ghetie V, Ward ES. Mapping the site on human IgG for binding of the MHC class I-related receptor, FcRn. *Eur J Immunol.* (1999) 29(9):2819–25. doi: 10.1002/(SICI)1521-4141(199909)29:09<2819::AIDIMMU2819>3.0.CO;2-6

58. Zhang B, Gollapudi D, Gorman J, O’Dell S, Damron LF, McKee K, et al. Engineering of HIV-1 neutralizing antibody CAP256V2LS for manufacturability and improved half-life. *Sci Rep.* (2022) 12(1):17876. doi: 10.1038/s41598-022-22435-2

59. Gollapudi D, Rosales-Zavala E, Ivleva VB, Yang Y, Li Y, O’Connell S, et al. Analytical characterization of broadly neutralizing antibody CAP256LS heavy chain clipping during manufacturing development. *Biotechnol Prog.* (2022) 38:e3296. doi: 10.1002/btpr.3296

60. Corey L, Gilbert PB, Juraska M, Montefiori DC, Morris L, Karuna ST, et al. Two randomized trials of neutralizing antibodies to prevent HIV-1 acquisition. *N Engl J Med.* (2021) 384(11):1003–14. doi: 10.1056/NEJMoa2031738

61. Brady JR, Love JC. Alternative hosts as the missing link for equitable therapeutic protein production. *Nat Biotechnol.* (2021) 39(4):404–7. doi: 10.1038/s41587-021-00884-w

62. Temrikar Z, Suryawanshi S, Meibohm B. Pharmacokinetics and clinical pharmacology of monoclonal antibodies in pediatric patients. *Paediatr Drugs.* (2020) 22(2):199–216. doi: 10.1007/s40272-020-00382-7
